# Identification of Smurf2 as a HIF-1α degrading E3 ubiquitin ligase

**DOI:** 10.18632/oncotarget.28081

**Published:** 2021-09-28

**Authors:** Shuai Zhao, Wafik S. El-Deiry

**Affiliations:** ^1^Laboratory of Translational Oncology and Experimental Cancer Therapeutics, Warren Alpert Medical School, Brown University, Providence, RI, USA; ^2^Pathobiology Graduate Program, Brown University, Providence, RI, USA; ^3^Department of Pathology and Laboratory Medicine, Brown University, Providence, RI, USA; ^4^Joint Program in Cancer Biology, Brown University and Lifespan Cancer Institute, Providence, RI, USA; ^5^Cancer Center at Brown University, Warren Alpert Medical School, Brown University, Providence, RI, USA; ^6^Hematology/Oncology Division, Lifespan Cancer Institute, Providence, RI, USA

**Keywords:** Smurf2, CDK4/6 inhibition, HIF1alpha, hypoxia, cancer therapy

## Abstract

The major adaptive response to hypoxia involves hypoxia-inducible factor HIF-1α which is regulated by von Hippel Lindau (VHL) E3 ligase. We previously observed a stabilization of HIF-1α by cyclin-dependent kinases CDK1 and CDK4/6 that is independent of VHL, hypoxia or p53, and found that CDK4/6 inhibitors destabilize HIF-1α under normoxia and hypoxia. To further investigate the molecular mechanism of HIF-1α destabilization by CDK1 or CDK4/6 inhibitors, we performed a proteomic screen on immunoprecipitated HIF-1α from hypoxic colorectal cancer cells that were either untreated or treated with CDK1 inhibitor Ro3306 and CDK4/6 inhibitor palbociclib. Our proteomics screen identified a number of candidates that were enriched in palbociclib-treated hypoxic cells including SMAD specific E3 ubiquitin protein ligase 2 (Smurf2). We also identified a HIF-1α peptide that appeared to be differentially phosphorylated after palbociclib treatment. Gene knockdown of *SMURF2* increased basal expression of HIF-1α even in the presence of Ro3306 or two different CDK4/6 inhibitors, palbociclib and abemaciclib. Overexpression of Smurf2 inhibited expression of HIF-1α and enhanced HIF-1α ubiquitination in normoxia. Proteasome inhibitor MG-132 partially rescued HIF-1α expression when Smurf2 was overexpressed. Smurf2 overexpression also inhibited HIF-1α expression level in two other cell lines, SW480 and VHL-deficient RCC4. Overexpression of *SMURF2* mRNA is correlated with improved disease-free survival and overall survival in clear cell renal cell cancer. Our results unravel a previously unknown mechanism involving Smurf2 for HIF-1α destabilization in CDK4/6 inhibitor-treated cells, thereby shedding light on VHL-independent HIF-1α regulation.

## INTRODUCTION

Angiogenesis in solid tumors often results in abnormal vasculature. The lack, leaking, distortion and occlusion of blood vessels impedes oxygen delivery. Oxygen consumption by uncontrolled tumor growth adds onto the oxygen deficiency. The intratumoral hypoxia creates a specific microenvironment that activates the adaptive responses mediated by hypoxia-induced factor 1α (HIF-1α). ΗIF-1α is the alpha subunit of HIF-1, the transcription factor that modulates the expression of a diverse group of genes that contribute to increasing oxygen delivery and metabolic accommodation to hypoxia. Under normoxic conditions, HIF-1α is hydroxylated, which recruits the von Hippel-Lindau (VHL) complex for polyubiquitination, subsequently prompting proteasomal degradation [1]. Under hypoxic conditions, the hydroxylation of HIF-1α is suppressed. HIF-1α accumulates and translocates into the nucleus. Upon heterodimerization with the HIF-1β subunit, it induces the transcription of various target genes, a number of which are biologically involved in cancer.

Enhanced HIF-1 signaling promotes vascularization to increase oxygen supply and facilitates the survival of malignant cells in adaptation to the hypoxic nature of cancer. It is also involved in metabolism alteration, immune evasion, cell invasion, and metastasis-initiating characteristics [2]. Overexpression of HIF-1α is observed in a variety of cancers and predicts unfavorable prognosis. There have been several attempts to therapeutically target HIF-1α through the blockade of its interaction with HIF-1β, interfering with its DNA binding affinity, disruption of its transcriptional activity, and inhibiting its mRNA and protein expression. Till now, development of therapies targeting HIF-1α remains hindered. Therefore, it is imperative to explore the mechanism of HIF-1α regulation in cancer cells and investigate new possibilities to therapeutically target HIF-1 signaling.

Previously we have described a non-canonical stabilization of HIF-1α by CDK1 in a VHL-independent manner, and further proposed CDK4 also as a HIF-1α stabilizer [3]. The mechanism of the regulation of HIF-1α by CDK4 is yet to be elucidated. To improve our understanding of HIF-1α stabilization by CDKs and gain novel insights into the mechanisms of HIF-1α regulation in cancer, we performed a proteomic analysis on immunoprecipitated HIF-1α from colorectal cancer cells treated in hypoxia. Among the proteins that were differentially present with palbociclib treatment compared to untreated control, the SMAD-specific E3 ubiquitin protein ligase 2 (Smurf2) was identified as a potential E3 ubiquitin ligase involved in HIF-1α destabilization.

As an E3 ubiquitin ligase, Smurf2 was originally known to regulate the TGF-β signaling pathway. Activation of the TGF-β signaling starts at ligand binding to the TGF-β type II receptors, which dimerize and transphosphorylate TGF-β type I receptors. The activated TGF-β type I receptors in turn phosphorylate regulatory SMAD proteins (R-Smads) (e.g., SMAD2 & SMAD3), which can then dimerize with the co-SMAD, SMAD4 [4]. The complex then translocates into the nucleus, interacts with other co-factors, binds target genes, and activates or represses transcription. In the nucleus, Smurf2 interacts with SMAD7 and translocates to the cytoplasm, where it targets the TGF-β receptor [5] as well as SMAD2 [6] and SMAD3 [7] for ubiquitination and degradation. Other examples of Smurf2 substrates include HSP27 (heat shock protein 27) [8], Yin Yang 1 (YY1) [9], Krüppel-like factor 5 (KLF5) [10], and poly(ADP-ribose) polymerase-1(PARP1) [11]. As a member of the HECT-type E3 ligases, Smurf2 contains a C2-WW-HECT structure. The C2 domain at N-terminal allows docking to the intracellular membranes. The WW domains are implicated in protein interactions. The HECT domain at the C-terminal is catalytic and is conserved among HECT-class E3 ligases. An interaction between C2 and HECT domains results in the Smurf2 autoinhibition [12].

Here we uncover and describe the novel role of Smurf2 in HIF-1α regulation and propose the mechanism whereby Smurf2 mediates the ubiquitination of HIF-1α which results in HIF-1α degradation upon CDK4/6 inhibition.

## RESULTS

### Identification of Smurf2 as a potential HIF-1α regulator

**Figure 1:**
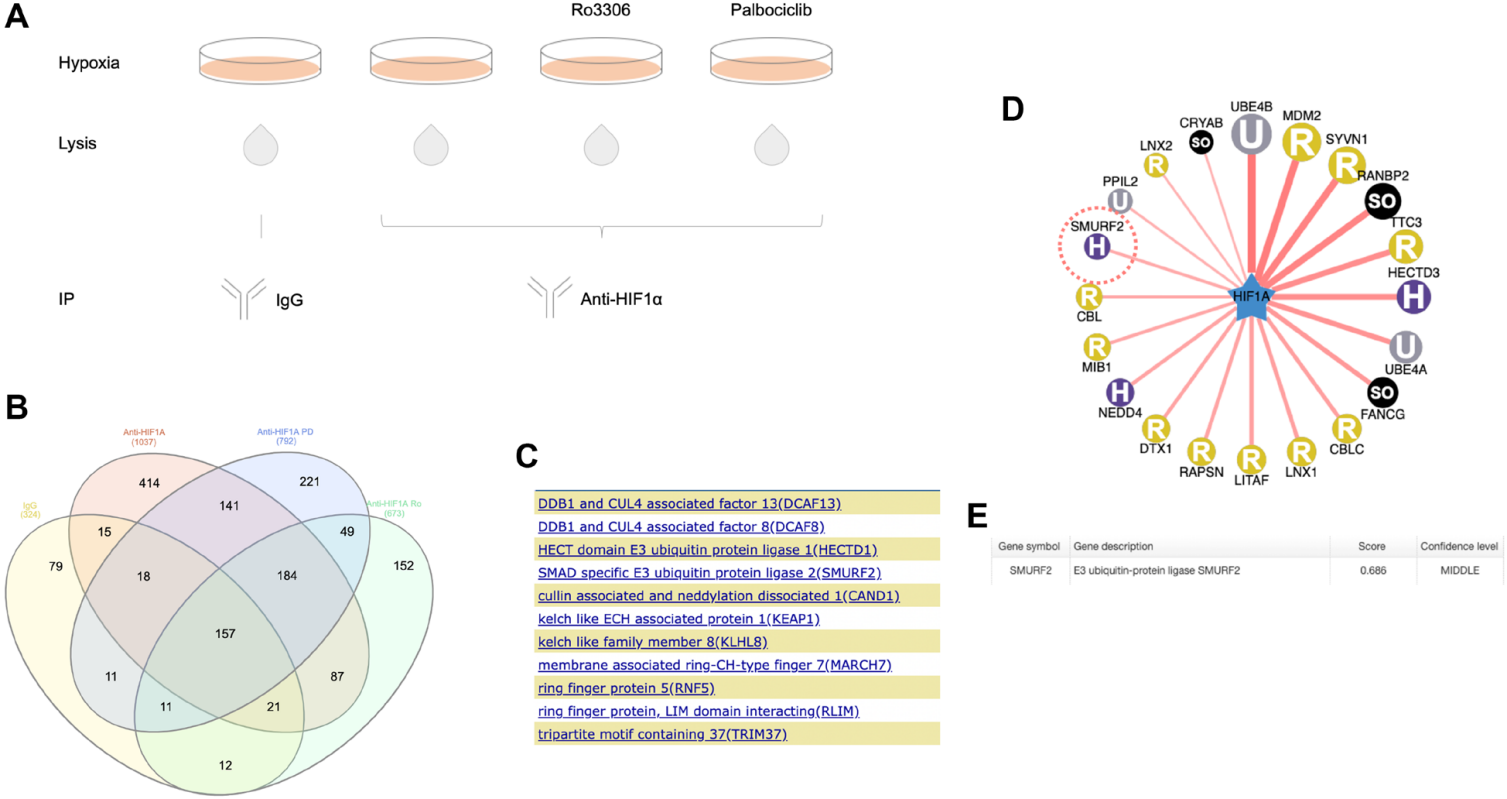
Proteomic analysis of immunoprecipitated HIF-1α from hypoxic colon cancer cells. (**A**) Schematic diagram showing the design of immunoprecipitation procedure. HCT116 cells were untreated or treated with Ro3306 or palbociclib in the presence of MG132 under hypoxia (0.5% O_2_) for 6 hours. Immunoprecipitation with HIF-1α antibody was performed with cell lysates from different treatment groups. IgG precipitation was used as a control. (**B**) Venn diagram of the proteomics result. Proteomic analysis was performed on elutes from HIF-1α immunoprecipitation. Shown in the diagram is the numbers of proteins that appeared in individual treatment groups. Ro: Ro3306. PD: palbociclib. The Venn diagram is generated using InteractiVenn. (**C**) Proteins appeared in palbociclib-treated but not untreated HIF-1α precipitates that are involved in the ubiquitination pathway. (**D**) A predicted HIF-1α-E3 ligase network with E3 ligases acting as single (instead of in a complex) at a confidence score between 0.671 and 0.781. The color and character in each circle represents the E3 ligase subtype. H: HECT; U: UBOX; SO: Single_other; R: RING. (**E**) Smurf2 was predicted to target HIF-1α at a confidence score of 0.686. The prediction was generated using the UbiBrowser tool (http://ubibrowser.ncpsb.org/).

Analysis on the HIF-1α co-precipitates (Figure 1A) identified 826 proteins in the anti-HIF-1α antibody but not IgG treated group (Figure 1B). In the previous study, knockdown of CDK4 decreased the expression of HIF-1α in RCC4 VHL-deficient cells [3], indicating that the stabilization of HIF-1α by CDK4/6 was independent of the VHL pathway. We hypothesized that there may be a E3 ubiquitin ligase, instead of VHL, that targets HIF-1α for ubiquitination upon CDK4/6 inhibition. We performed a gene-set enrichment analysis on the 270 proteins appeared in the palbociclib (CDK4/6 inhibitor) treated but not untreated or IgG group (Figure 1B). Among them, 11 proteins were identified in the Ubl (ubiquitin-like) conjugation pathway (Figure 1C), including an E3 ubiquitin ligase, Smurf2 (SMAD Specific E3 Ubiquitin Protein Ligase 2).

### Prediction of Smurf2 as a HIF-1α-targeting E3 ubiquitin ligase

At the same time, we also identified this protein in a bioinformatic prediction of E3 ligases that may target HIF-1α (Figure 1D). The prediction is based on a Bayesian network that was developed by computationally analyzing the existing E3-substrate pairs and evaluating the potential evidence such as E3-substrate interacting domains, GO term enrichment, protein interaction network topology, E3 recognizing motifs and ortholog interactions [13].

Smurf2 appeared at a middle confidence level with a confidence score of 0.686 (Figure 1E) (a likelihood ratio of 6.08), which is even higher than that (0.632) (a likelihood ratio of 3.47) in the prediction of Smurf2 as a E3 ligase for the enhancer of zeste homolog 2 (EZH2), with the latter already demonstrated as a substrate of Smurf2 [14]. This supported the hypothesis that Smurf2 is a E3 ubiquitin ligase that targets HIF-1α.

### Smurf2 interacts with HIF-1α protein

**Figure 2:**
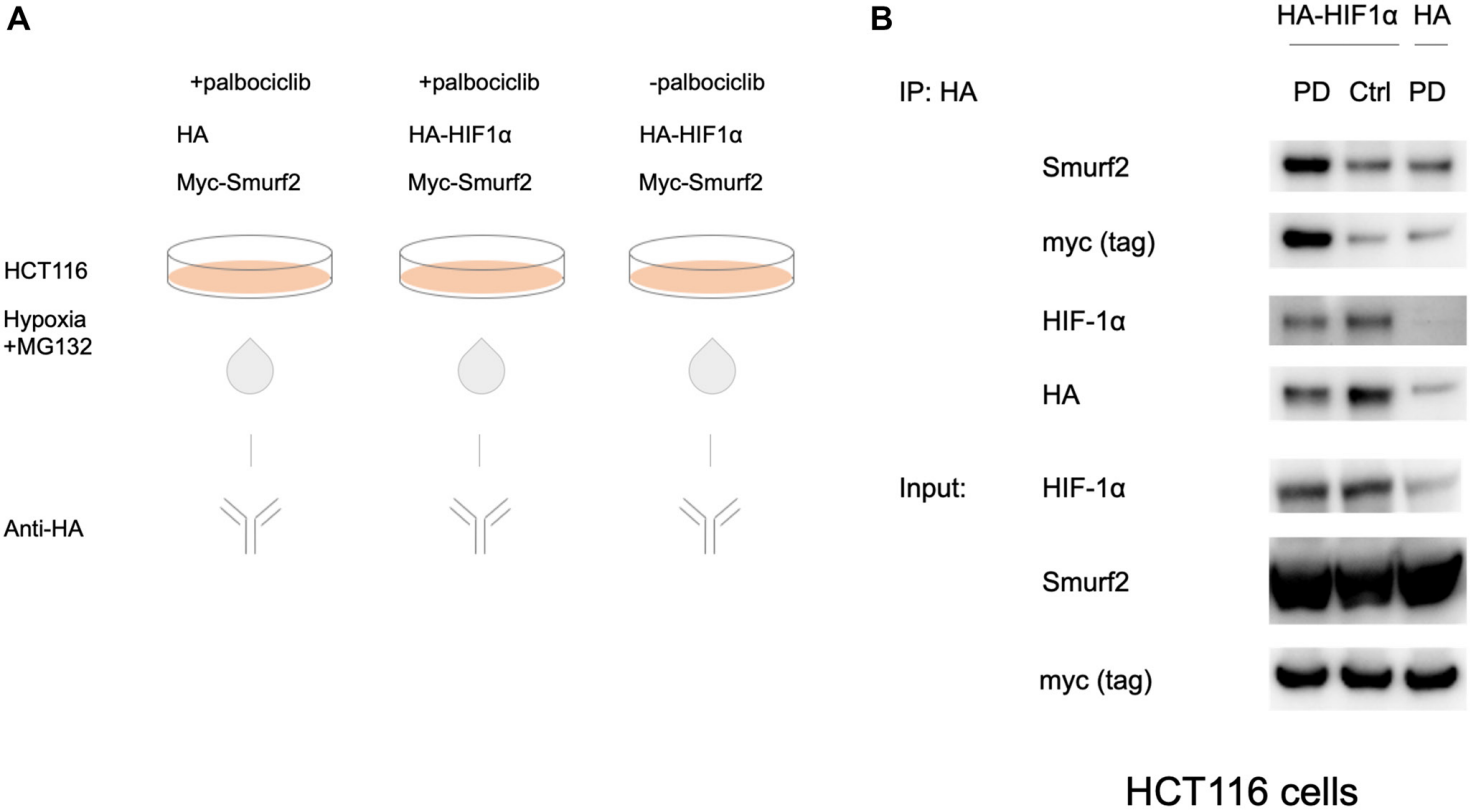
Interaction between HIF-1α and Smurf2. (**A**) HCT116 cells were transfected with the HA-HIF-1α-overexpressing plasmid or the HA-tag-containing control vector for 24 hours. Subsequently the cells were treated with or without palbociclib under hypoxia for 6 hours in the presence of 10 μM MG132. Anti-HA antibody was used for immunoprecipitation. (**B**) Co-immunoprecipitation of HA-HIF-1α and myc-Smurf2 with palbociclib treatment.

To confirm the interaction between Smurf2 and HIF-1α, we performed a co-immunoprecipitation assay in the HCT116 colorectal cancer cell line. HCT116 cells were co-transfected with an HA-HIF-1α-overexpressing plasmid or an HA-tag-containing control vector together with an myc-Smurf2-expressing plasmid (Figure 2A). After 24 hours, the cells were treated with or without the CDK4/6 inhibitor palbociclib under hypoxia for 6 hours. MG132 was added to inhibit proteasomal degradation and preserve protein expression. A co-immunoprecipitation experiment was performed with anti-HA antibody to precipitate HA-HIF-1α. Anti-Smurf2 antibody and anti-myc tag antibody were used to detect the co-precipitated myc-Smurf2 protein.

The exogenous expression of HA-HIF-1α increases the level of HIF-1α protein under hypoxia (Figure 2B). Treatment with palbociclib promotes the interaction between HIF-1α and Smurf2 as compared to the control group without palbociclib treatment or the HA-tag-expressing group in which the background-level detection was likely due to non-specific binding. This result confirms the role of Smurf2 as a HIF-1α-interacting partner and prompted us to test Smurf2-mediated effects on HIF-1α regulation.

### Smurf2 regulates HIF-1α expression

**Figure 3:**
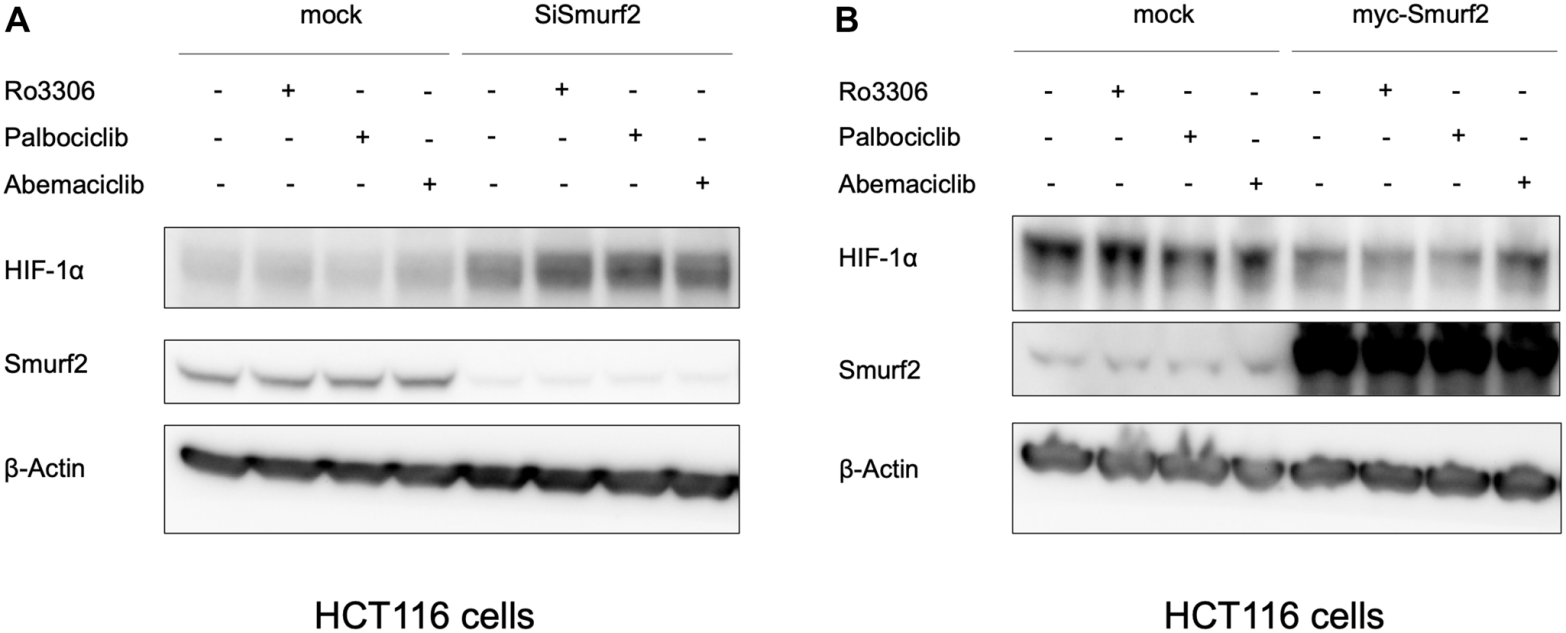
Smurf2 regulates HIF-1α expression in HCT116 colon cancer cells. (**A**) Knockdown of Smurf2 increases HIF1α expression in normoxia, shown by western blot. Cells were transfected with Smurf2 siRNA for 48 hours and treated with indicated reagents in normoxia for 6 hours. Ro3306: 5 μM; palbociclib: 10 μM; abemaciclib: 1 μM. (**B**) Overexpression of Smurf2 decreases the expression of HIF-1α in hypoxia (0.5% O_2_). Cells were transfected with plasmids overexpressing myc-Smurf2 for 24 hours and treated with indicated reagents in hypoxia for 6 hours.

**Figure 4:**
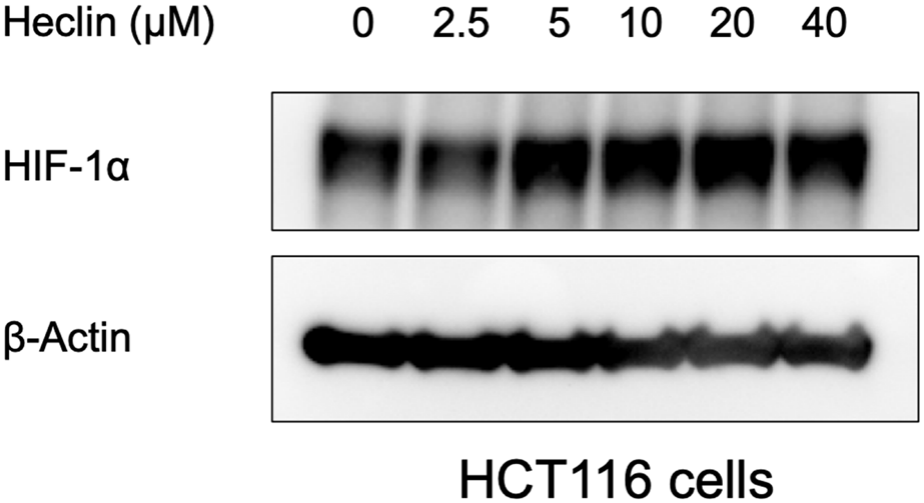
Heclin treatment elevates HIF-1α expression levels. HCT116 cells were treated with Heclin at the indicated doses for 6 hrs in hypoxia, and western blotting was performed.

The proteomic analysis and bioinformatic prediction on E3 ligase - substrate interactions together suggested a potential for Smurf2 to be involved in HIF-1α destabilization when cells are treated with a CDK4/6 inhibitor. To test whether Smurf2 affects the level of HIF-1α, we transfected the HCT116 colorectal cancer cells with a Smurf2-targeting siRNA to decrease its expression. Knockdown of Smurf2 increased HIF-1α expression in normoxia (Figure 3A), which was not reversed by treatment with two distinct CDK4/6 inhibitors, palbociclib and abemaciclib, or a CDK1 inhibitor, Ro-3306. Overexpression of Smurf2 with a plasmid bearing myc-tagged *SMURF2* sequence decreased the expression of HIF-1α in hypoxia (Figure 3B). We also tested another colorectal cancer cell line, SW480, in which overexpression of Smurf2 similarly reduced HIF-1α expression level under hypoxia. Moreover, treatment with Smurf2 inhibitor, Heclin, increased the expression of HIF-1α in hypoxia (Figure 4). Our results suggest the possibility that Smurf2 targets HIF-1α and acts as an E3 ubiquitin protein ligase, which is involved in the HIF-1α destabilization upon inhibition of CDK4/6.

### Smurf2 promotes HIF-1α ubiquitination

To test the role of Smurf2 related to its E3 ubiquitin ligase activity, we performed a ubiquitination assay on HIF-1α. HCT116 cells were co-transfected with plasmids containing HA-ubiquitin and Smurf2-targeting siRNA or a Smurf2-overexpressing plasmid. After 2 days, MG132 was used in pretreatment of the cells for 30 min, followed by addition of the treatment with or without palbociclib. MG132 is a proteasome inhibitor and was included to inhibit ubiquitination-mediated protein degradation. Cells were harvested at 4 hours and lysed in NP-40 lysis buffer containing N-ethylmaleimide with preservation of the ubiquitinated species. Anti-HIF-1α antibody was used to immunoprecipitate HIF-1α. Ubiquitination was detected by probing for the HA tag on ubiquitin in the precipitates.

**Figure 5:**
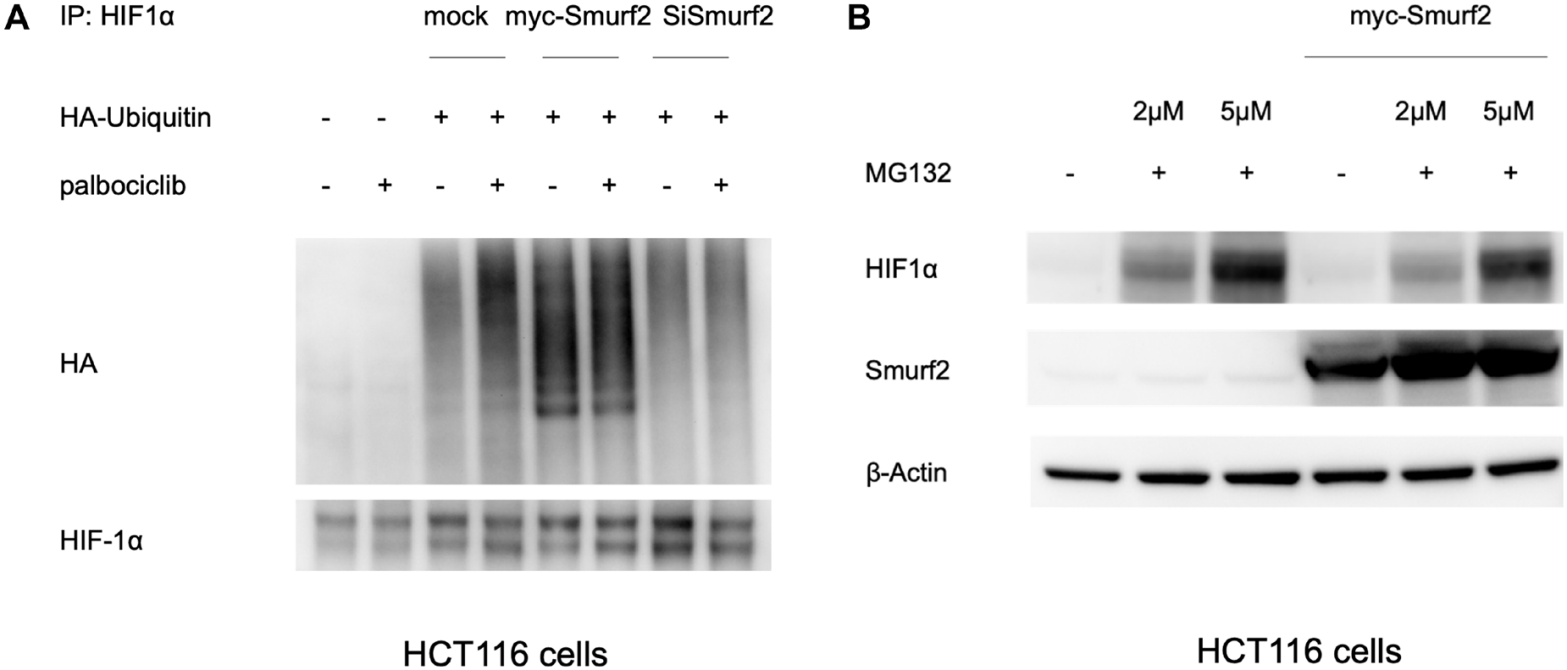
Smurf2 enhances HIF1α ubiquitination in normoxia. (**A**) Ubiquitination analysis on HIF-1α with the overexpression or knockdown of Smurf2. HCT116 cells were co-transfected with plasmids expressing HA-Ubiquitin together with myc-Smurf2 or Smurf2 siRNA for 48 hours and treated with or without palbociclib in presence of MG132 in normoxia for 4 hours. Cell lysates were immunoprecipitated with HIF-1α antibody. Ubiquitination was indicated by HA staining in western blot. (**B**) Proteasome inhibition partially rescues the HIF-1α expression upon Smurf2 overexpression. HCT116 cells were transfected with or without myc-Smurf2-overexpressing plasmid and subsequently treated with MG132 at indicated concentrations for 6 hours.

As a background control, HA was barely detected in the precipitates from cells without the ubiquitin transfection, compared to those with exogenously expressed HA-ubiquitin, indicating the specificity of the assay (Figure 5A). The amount of HA-ubiquitin ligated to HIF-1α was increased upon palbociclib treatment, which is in line with the expectation that CDK4/6 inhibition destabilizes HIF-1α through increasing its ubiquitination. Overexpression of Smurf2 remarkably elevated HIF-1α ubiquitination in normoxia with or without palbociclib, while knockdown of Smurf2 ablated the induced ubiquitination upon palbociclib treatment. Our results suggest that Smurf2 targets HIF-1α and induces its ubiquitination, which mediates the HIF-1α destabilization by palbociclib.

In addition, we tested the effect of proteasome inhibition on the level of HIF-1α upon Smurf2 overexpression. As expected, MG132 treatment increased HIF-1α expression at 2 μM and more robustly at 5 μM under normoxia (Figure 5B). With Smurf2 overexpression, MG132 partially rescued the HIF-1α expression in a dose-dependent manner. It is likely that Smurf2-induced HIF-1α suppression is mediated at least partially through proteasomal degradation of ubiquitinated HIF-1α.

### Smurf2 overexpression reduces HIF-1α level in RCC4 cells and SW480 cells

**Figure 6:**
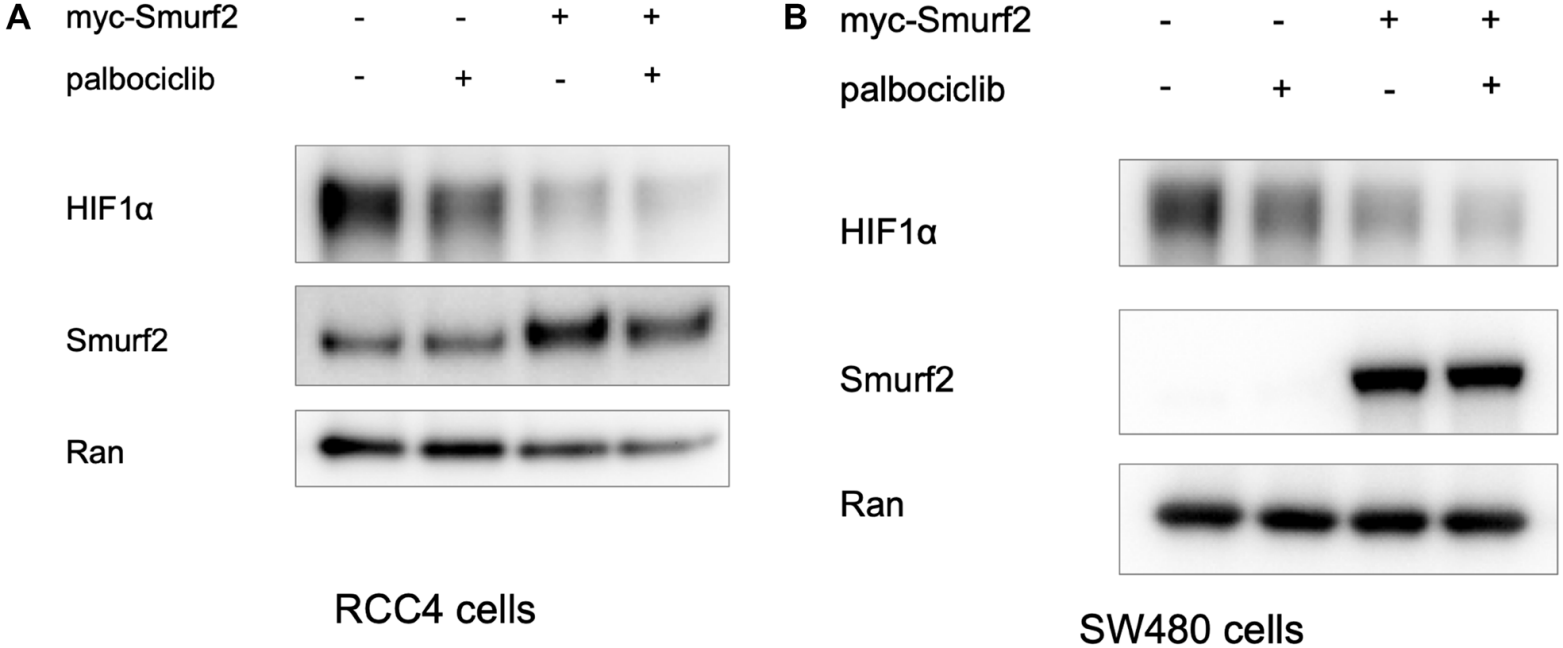
Overexpression of Smurf2 reduces HIF-1α expression level in RCC4 cells under normoxia and in SW480 cells under hypoxia. (**A**) The VHL-deficient renal cell cancer RCC4 cell line was transfected with a Smurf2-overexpressing plasmid for 24 hours and subsequently treated with or without palbociclib in normoxia for 6 hours. (**B**) SW480 cells were transfected with myc-Smurf2-overexpressing plasmid for 24 hrs and treated with or without palbociclib in hypoxia for 6 hours.

In clear cell renal cell carcinoma, *VHL* loss-of-function mutations frequently leads to VHL deficiency and hence the upregulation of HIF-1α protein expression regardless of oxygen concentration. We transfected the RCC4 clear cell renal cell carcinoma line with myc-Smurf2-overexpressing plasmid. The exogenous overexpression of Smurf2 decreased HIF-1α level in RCC4 cells in normoxia (Figure 6A). The destabilization effect on HIF1α by Smurf2 overexpression was also observed in SW480 cells under hypoxia (Figure 6B). The results reinforce our conclusion regarding the ability of Smurf2 to degrade HIF-1α and suggests a potential protective role of Smurf2 in clear cell renal cell carcinoma.

### High *SMURF2* expression is associated with good prognosis in KIRC

**Figure 7:**
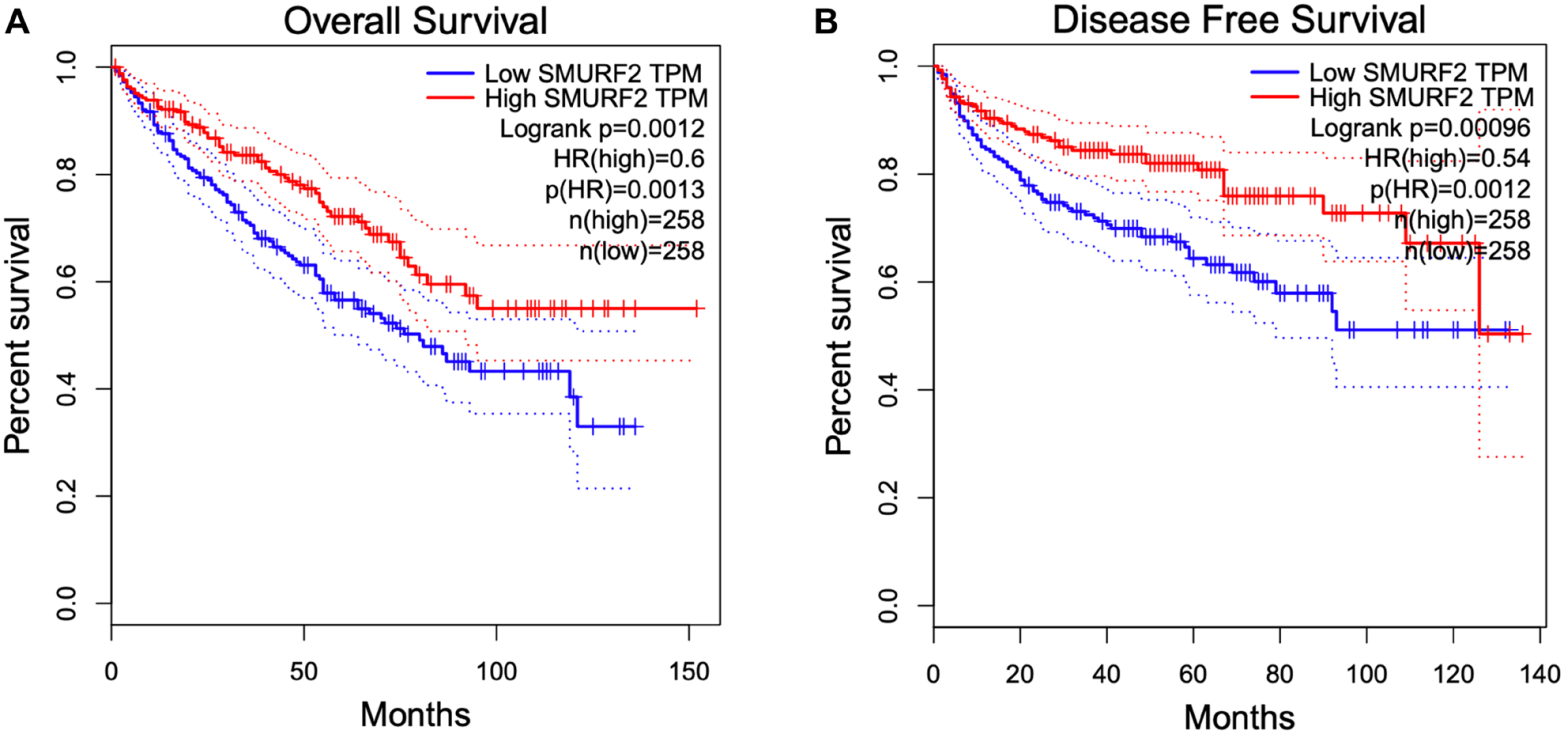
Overexpression of SMURF2 is associated with better prognosis in KIRC (kidney renal clear cell carcinoma). (**A**) Overall survival and (**B**) Disease-free survival in correlation to SMURF2 expression. Kaplan-Meier plots are generated with the GEPIA online analysis tool based on TCGA data using median as group cutoff. HR: hazard ratio.

Analysis of the TCGA data showed a positive correlation between *SMURF2* overexpression and increased overall survival and disease-free survival in kidney renal clear cell carcinoma (KIRC) (Figure 7). The clear cell renal cell cancer (ccRCC) cells frequently lack functional VHL. Deletion, mutation or epigenetic silencing of the *VHL* gene occurs in over 80% of ccRCC cases [15]. The most well characterized role of VHL is to recognize hydroxylated HIF-α as the substrate for ubiquitination by the E3 ubiquitin ligase complex in normoxia. VHL deficiency results in the accumulation of HIF-α subunits. It would be intriguing to explore whether Smurf2 plays a tumor suppressive role alternatively to VHL in this context through targeting HIF-α.

## DISCUSSION

We discovered a novel mechanism of regulation of HIF-1α, involving Smurf2 E3 ubiquitin ligase, in CDK4/6 inhibitor treated cancer cells where HIF-1α is destabilized.

Adaptation to deoxygenation is an important process in various physiological and pathological conditions. HIF-1α is a main mediator in hypoxic responses and plays a critical role in promoting angiogenesis. In a variety of cancers, hypoxia arises heterogeneously in a solid tumor mass, and HIF-1α overexpression contributes to cell survival, invasiveness and metastasis as well as chemo- and radio- therapy resistance. Targeting HIF-1α therefore has promising therapeutic potential in cancer treatment.

**Figure 8:**
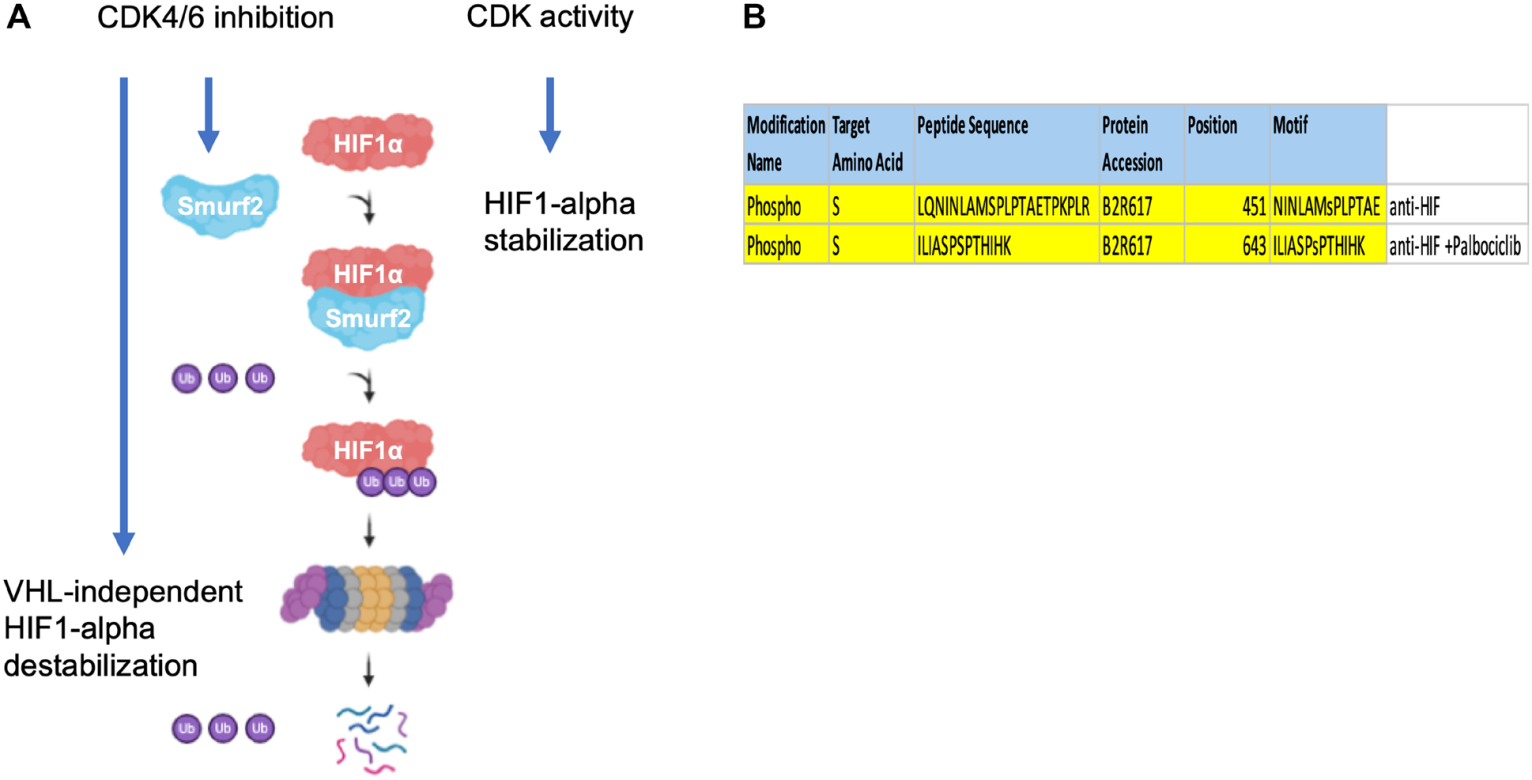
Proposed model of Smurf2-mediated HIF-1α regulation. (**A**) Smurf2 interacts with HIF-1α and targets it for ubiquitination, which results in HIF-1α destabilization. (**B**) Distinct HIF-1α phosphorylation peptides revealed by proteomics in control and palbociclib-treated groups.

Here we propose a non-canonical molecular mechanism where Smurf2 acts as an E3-ubiquitin ligase that targets HIF-1α for ubiquitination and destabilization upon inhibition of CDK4/6 (Figure 8A). There are several directions for future investigation.

In subsequent studies, it would be essential to test the involvement of Smurf2 in HIF-1α regulation in various cancer types especially including both VHL-sufficient and VHL-deficient cancers. Also, the interaction between HIF-1α and Smurf2 needs additional characterization. Existing studies have suggested that Smurf2 recognizes a PPxY or a LPxY motif in its substrates where PPxY occurs in more cases. This sequence is absent in HIF-1α protein. However, such motif appears to be dispensable in other established Smurf2 targets. For example, examination of EZH2, the previously identified Smurf2 substrate, has revealed an absence of the PPxY or LPxY region. A very recent investigation has found that only half of the WW-domain mediated interactions are based on the PY motifs [16]. Therefore, characterization of the Smurf2 recognition site located in HIF-1α and the type of ubiquitination it induces would improve the understanding of HIF-1α regulation mechanisms as well as unravel how Smurf2 selects substrates and exerts its ligase activity. Noticeably, we have observed a Ser451 phosphorylation on HIF-1α that appeared in the untreated but not the palbociclib-treated group (Figure 8B). It would be interesting to determine whether CDK4/6 directly phosphorylates HIF-1α at the Ser451 residue and to assess the role of such phosphorylation in maintaining HIF-1α stability. In addition, the mechanism by which Smurf2 potentiates HIF-1α degradation awaits further investigation. It would be useful to test whether Smurf2 affects HIF-1α transcription as well as to measure whether the lysosome is involved in Smurf2-induced HIF-1α degradation. It has been reported that the K63 (instead of K48) ubiquitination of HIF-1α mediates its chaperone-mediated autophagy, which leads to lysosomal degradation [17, 18].

Furthermore, considering the sequence homology, structure similarity and oxygen-dependent destabilizing pathways in common, HIF-2α represents a candidate aside from HIF-1α to be tested as a Smurf2 substrate. Indeed, using the bioinformatic tool, Smurf2 is predicted to act as a HIF-2α regulator at a middle confidence score as well. Although HIF-2α also does not contain a PY motif, investigation of HIF-2α ubiquitination may reveal the shared or distinct substrate recognition patterns with HIF-1α in Smurf2 activity.

Apart from providing new possibilities to target HIF signaling in cancer, exploring the novel HIF-α regulation mechanisms potentially contributes as well to other diseases with disrupted blood supplies. In these circumstances, HIF serves as a protective factor with its function in maintaining oxygen homeostasis through vascularization induction and metabolic programming. Targeting the HIF-α destabilization mechanisms has potential applications when boosting HIF signaling is preferred to achieve organ protection, such as in ischemic cardiovascular disease, lung and liver injuries, as well as chronic kidney diseases [19]. In particular, PHD inhibitors have shown therapeutic benefits in the treatment of chronic kidney diseases, some of which have acquired global approvals already [20, 21].

In summary, we propose a non-canonical mechanism involving Smurf2 in HIF-1α degradation upon CDK4/6 inhibitor treatment, which provides novel insights in HIF-1α regulation. It sheds light on the HIF-1α stabilization in cancer as well as suggests new possibilities of therapeutic angiogenesis.

## MATERIALS AND METHODS

### Cell culture

HCT116 and SW480 cells were obtained from American Type Culture Collection (ATCC). RCC4 cells were a generous gift from Dr. Celeste Simon (University of Pennsylvania). HCT116 cells were maintained according to ATCC recommendation in McCoy’s 5A medium (Hyclone) with 10% fetal bovine serum (FBS) (Hyclone) and 1% penicillin/streptomycin (P/S) (Corning). SW480 and RCC4 cells were maintained in DMEM medium with 10% FBS and 1% P/S. Cells were regularly tested for mycoplasma and authenticated. Cells were maintained at 37°C in 5% CO_2_. Hypoxia treatment was performed in a hypoxia chamber (*In vivo*2, Ruskinn) which maintains 0.5% O_2_ and 5% CO_2_ at 37°C.

### Antibodies

HIF-1α and Ran antibodies were purchased from BD Biosciences. HA and Smurf2 antibodies were purchased from Cell Signaling Technology. Actin antibody was purchased from Sigma.

### Reagents

MG-132 was purchased from Sigma. Ro-3306 was purchased from Santa Cruz Biotechnology. Palbociclib (PD-0332991, in form of palbociclib hydrochloride) and abemaciclib (in form of abemaciclib mesylate) were purchased from Medkoo Biosciences.

### Plasmids and siRNA

pcDNA3-HA-ubiquitin plasmid was from Edward Yeh lab (Addgene plasmid #18712). pRK-myc-Smurf2 plasmid was from Ying Zhang lab (Addgene plasmid #13678). Smurf2-targeting siRNA was purchased from Santa Cruz Biotechnology.

### Cell transfection

Plasmid expression was performed by 24 hours transfection using Opti-MEM (Thermo Fisher Scientific) and Lipofectamine 2000 (Life Technologies). Knockdown experiments were performed by siRNA transfection for 48 hours with Opti-MEM and Lipofectamine RNAiMAX (Life Technologies), according to the manufacturer’s protocol.

### Western blot

Treated cells were lysed in RIPA buffer (Sigma). Protein concentrations were determined using a BCA Protein Assay Kit (Life Technologies). Equal amounts of total protein were boiled with NuPAGE^™^ LDS sample buffer (Thermo Fisher Scientific) and 2-Mercaptoethanol. Samples were analyzed with SDS-PAGE. Proteins were transferred to an Immobilon-P PVDF membrane (EMD Millipore). Primary and secondary antibodies were added in order. Signals were detected after addition of the ECL western blotting substrate (Thermo Fisher Scientific).

### Immunoprecipitation

HCT116 cells were cultured in hypoxia with or without treatment of Ro3306 or palbociclib for 6 hours in the presence of MG132 (2 μM), washed with PBS, and harvested and lysed in NP40 cell lysis buffer (Thermo Fisher Scientific) containing protease inhibitor cocktail (Roche) and phosphatase inhibitor cocktail (Roche). Cell debris was removed after centrifugation at 13,200 rpm in 4°C. Protein concentration was measured with BCA assay. The amounts of protein were equalized among treatments. Lysates was incubated with anti-HIF-1α antibody overnight at 4°C, followed by precipitation with Protein A/G Ultra link Resin (Thermo Fisher Scientific) for 2–4 hours. After precipitation, the resin was washed with NP-40 buffer for four times according to manufacturer’s protocol. Precipitated proteins were eluted using 8M urea buffer. Elutes were collected from repeated elution to ensure the maximum release of captured proteins from resin.

### Proteomic mass spectrometry

Proteomic mass spectrometry on IP elutes was performed by the Proteomics core facility at COBRE Center for Cancer Research Development at Rhode Island Hospital. Proteins precipitated in individual treatment groups were compared to identify the difference in interaction patterns.

### Analysis on the proteomics

Venn diagram was generated using the InteractiVenn software (http://www.interactivenn.net/), which shows the number of proteins determined by proteomics in precipitates from each treatment set. The differentially presented proteins were analyzed with functional annotation clustering tool at DAVID Bioinformatics Resources (https://david.ncifcrf.gov/).

### E3 ubiquitin ligase prediction

The bioinformatic prediction of E3-ligases was performed with aid of the UbiBrowser tool (http://ubibrowser.ncpsb.org/) [13].

### Ubiquitination assay

HCT116 cells were co-transfected with plasmids overexpressing HA-ubiquitin together with either myc-Smurf2-expressing plasmid or Smurf2 siRNA (or neither as a control). After 48 hours of transfection, the cells were pretreated with MG-132 for 30 min prior to addition of the treatment with or without palbociclib under normoxia for 4 hours, and then harvested on ice in NP40 cell lysis buffer with 10 mM N-ethylmaleimide (Sigma-Aldrich) as an isopeptidase inhibitor to preserve the ubiquitination. Cell lysates were centrifuged at 13,200 rpm for 20 min. The supernatants were incubated with anti-HIF-1α antibody overnight at 4°C. Protein A/G Ultra link Resin was added and allowed binding for another 2–4 hours. After washing for 4 times, the beads were resuspended in LDS loading buffer and boiled to release and denature the proteins. The elutes were analyzed in western blot.

### Correlation analysis

The patient outcome analysis (Kaplan-Meier plot) was performed with the GEPIA web server (http://gepia.cancer-pku.cn/) based on TCGA KIRC (Kidney renal clear cell carcinoma). Median was used as group cutoff. Hazard ratio (HR) was calculated.
